# Neglected mpox resurges with virulence in Africa: Will this be another neglected warning?

**DOI:** 10.4102/jphia.v15i1.730

**Published:** 2024-08-16

**Authors:** Oyewale Tomori

**Affiliations:** 1 African Centre of Excellence for Genomics of Infectious Diseases, Redeemer’s University, Ede, Osun State, Nigeria

Monkeypox, now known as the human mpox, is an infectious disease caused by monkeypox virus (MPXV), which is endemic and was first reported in the 1970s in the regions of West, Central and East Africa, particularly in the densely forested northern and central regions of the Democratic Republic of the Congo.^1^

There are two clades of MPXV strain: Clade I, formerly known as the Congo Basin or Central African clade) and Clade II, formerly known as the West African clade).^2^ Clade II is subdivided into two subclades, IIa and IIb, the latter being responsible for the 2022–2024 ongoing multi-country outbreak. Historically, Clade I predominates in the Democratic Republic of Congo (DRC) and accounts for 95% of reported cases, while Clade II has been active in the West African region.^2^

The response of governments of mpox endemic countries and global health authorities to the over 50 years of mpox outbreaks in Africa has been consistently poor and ineffective.^3^ However, in 2022, a new phase of MPX began when the first case of the disease, not associated with travel from Africa, was reported in the UK. This triggered the multi-country MPX outbreak,^4^ which the WHO declared a global public health emergency of international concern (PHEIC) in July 2022. By May 2023, when the PHEIC was declared over, 118 countries (7 mpox endemic and 111 mpox non-endemic) had reported a total of 87 377 cases (1587 in endemic and 87 377 in non-endemic countries).^5^

However, since 2023, there has been an ongoing outbreak with dramatic increase in the number of mpox cases reported in the DRC.^1^ In 2023, DRC reported a total of 14 434 suspected mpox cases with 728 deaths. In 2024 (as of 29 March), 4488 suspected cases, with 319 confirmed, have been reported. A total of 279 deaths have been reported in the country in 2024 case fatality rate [CFR]: 6.2%). Mpox cases have been reported in 23 of 26 provinces of the DRC.^6^ There is a notable increased human-to-human transmission, with children ≤15 years representing 67% of suspected cases and 84% of the deaths.^4^ Children under the age of 1 are reported to be four times more likely to die compared to those over the age of 15 years. Available but limited data show that the most reported symptoms were fever 38/51 (75%) and rash 45/51 (88%). Among those with a rash, the majority had oral and anogenital lesions. Mpox viral DNA was detected by qPCR from vaginal, penile and oral swabs in over 70% of individuals.^7^

Another intriguing development about Mpox in DRC, is that, before April 2023, no cases of sexual transmission of clade I MPXV have been formally documented. The first known cases were reported when a Belgium resident with connections to the DRC, tested positive for clade I MPXV in Kenge, Kwango province, during a visit to the DRC.^8^ Thereafter, his sexual contacts in the DRC also tested positive for clade I MPXV, with closely related viral sequences. This is the first reported clade I MPXV infection linked to sexual transmission within a cluster. Another outbreak in the country is also being reported with multiple cases of mpox among sex workers.^8^

The recent resurgence of monkeypox in the DRC^1,6^ is indeed a concerning issue that warrants swift and decisive action. There has always been an urgent but unattended need to address several poorly understood issues and factors contributing to the endemicity of mpox in Africa and the current resurgence of the disease in DRC. One of these factors is the close interaction between humans and animals in many African rural communities, providing opportunities for the virus to spread from animals to humans. Additionally, deforestation and climate change factors that alter the habitats of the MPXV animal reservoirs may also play a role in increasing human–animal contact and consequent risk of exposure leading to outbreaks.

To address the resurgence of Mpox in DRC and increased risk of spread to other countries, the following measures need to be taken:

- *Public Awareness and Education*: Educating communities about the transmission of mpox and promoting preventive measures such as hand hygiene, avoiding contact with sick animals and seeking medical care promptly can help reduce the risk of transmission.
- *Enhanced Surveillance*: Surveillance systems must be strengthened for early detection of cases to contain outbreaks. Providing specific case definitions for suspected monkeypox cases and integrating mpox surveillance into existing disease surveillance systems would improve the timely reporting of suspected cases and the initiation of rapid response and control activities. In addition, participatory community (mpox) surveillance will positively impact early detection of cases.
- *Improved Laboratory Diagnosis*: Improving mpox diagnosis is crucial for effective outbreak management and control. The capacity for rapid and accurate laboratory diagnosis must be strengthened through providing laboratories with the necessary tools, reagents and expertise to conduct diagnostic tests, such as polymerase chain reaction (PCR) assays and serological testing. Sensitive, specific, affordable and easy-to-use mpox rates must be developed and deployed for use by healthcare workers at the point-of-care.
- *Cross-Border Collaboration*: Monkeypox outbreaks in Africa often cross national and international borders, highlighting the need for regional collaboration and information sharing. Establishing mechanisms for cross-border collaboration, such as joint surveillance activities, data sharing agreements and coordinated response efforts, can enhance the effectiveness of field epidemiology interventions.
- *Integration with One Health Approaches*: By recognising the interconnectedness of human, animal and environmental health, mpox transmission and spread will be better understood, making it easier to control outbreaks. Consequently, integrating field epidemiology activities using One Health approach is essential for monitoring animal reservoirs, identifying high-risk areas and implementing targeted interventions.
- *Research and Development* (*R&D*): It is essential to invest in R&D to improve existing diagnostic tools and develop new technologies for mpox diagnosis. Funding must be provided for the development of novel diagnostic assays, point-of-care devices and other innovative approaches to enhance mpox diagnosis.

The poor knowledge about the natural hosts and or reservoirs of mpox calls for adequate and sustained funding support by natural hosts and or reservoirs African governments for African scientists to conduct relevant research to unravel the diversity of mpox natural hosts/reservoir and elucidate the modes of animal–human transmission.

- *Vaccines and Vaccination*: Vaccination is an important tool in stopping the spread of mpox. JYNNEOS is a two-dose vaccine developed to protect against mpox and smallpox. For best protection, both doses are required, with the second dose should be given 4 weeks after the first dose. There is an urgent need for governments to procure the vaccines for use in controlling the outbreak.

From 11 to 13 April 2024, the African CDC organised a *High-Level Emergency Regional Meeting on mpox in Africa* in Kinshasa from 11 to 13 April 2024. The meeting in recognising the need for the effective control of mpox, called on Member States and institutions to DECIDE to establish the Africa Taskforce for mpox. The outcome of this high-level meeting falls far short of expectations. Given that it has been over 50 years as the first mpox case was reported in Africa, African governments and scientists should have moved beyond deciding to set up a mpox Task Force to implementing agreed plans of action. African governments must go beyond mere political words to addressing the grave issues of mpox and other disease outbreaks. Africa must move from dependence on foreign donors and provide adequate and sustained national financial resources for disease prevention and control activities. The governments must create an enabling and conducive local environment for scientists and researchers to function maximally and effectively. This will empower African scientists and researchers to provide evidence-based scientific data to guide national policy. Investment of human and financial resources in addressing emerging and re-emerging disease outbreaks will yield high returns on investment on improved public health and socio-economic well-being. National investment in health R&D will yield huge returns of investments for securing national health security.
